# Milk of Cow and Goat, Immunized by Recombinant Protein Vaccine ZF-UZ-VAC2001(Zifivax), Contains Neutralizing Antibodies Against SARS-CoV-2 and Remains Active After Standard Milk Pasteurization

**DOI:** 10.3389/fnut.2022.901871

**Published:** 2022-06-13

**Authors:** Victoria Garib, Stefani Katsamaki, Shahlo Turdikulova, Yuliya Levitskaya, Nodira Zahidova, Galina Bus, Kristina Karamova, Manona Rakhmedova, Nigora Magbulova, Alexander Bruhov, Firuz Y. Garib, Ibrokhim Y. Abdurakhmonov

**Affiliations:** ^1^Division of Immunopathology, Department of Pathophysiology and Allergy Research, Medical University of Vienna, Vienna, Austria; ^2^International Centre of Molecular Allergology, Ministry of Innovative Development of the Republic of Uzbekistan, Tashkent, Uzbekistan; ^3^Centre of Advanced Technology, Ministry of Innovative Development of the Republic of Uzbekistan, Tashkent, Uzbekistan; ^4^Scientific and Diagnostical Centre of Laboratory Technology “Defactum Laboratories”, Tashkent, Uzbekistan; ^5^Centre of Genomics and Bioinformatics, Academy of Sciences of Uzbekistan, Tashkent, Uzbekistan

**Keywords:** bovine immunoglobulins, SARS-CoV-2, immune milk, neutralizing antibody (nAb), passive immunization, vaccination, ZF-UZ-VAC2001

## Abstract

Here, we present the first experimental validation of the possibility for obtaining immune milk with neutralizing antibodies against SARS-CoV-2 from vaccinated cows and goat using approved recombinant protein human coronavirus vaccine, ZF-UZ-VAC2001, in the Republic of Uzbekistan. In the period of 2 weeks after first vaccination, we detected the neutralizing antibodies against coronavirus in the blood serum of vaccinated animals. The neutralizing activity, in its peak on the 21st day after receiving the third dose (77th day from first dose), was effective in neutralization test using a live SARS-CoV-2 in Vero E6 cells, even after 120-fold serum titration. In cows receiving three dose of human vaccine, the MAGLUMI^®^ SARS-CoV-2 neutralizing antibody competitive chemiluminescence immunoassay revealed that colostrum of the first day after calving had a greater activity to neutralize the SARS-CoV-2 compared to colostrum of subsequent three days (4.080 μg/ml vs 2.106, 1.960 and 1.126 μg/ml). In comparison, the neutralizing activity for goat and cow milk was 1.486 μg/ml and 0.222 μg/ml, respectively. We observed a positive correlation of receptor-binding domain (RBD)-specific IgG antibodies between the serum of actively immunized cow and milk-feeding calf during the entire course of vaccination (*r* = 0.95, *p* = 0.05). We showed an optimal regime for immune milk pasteurization at 62.5°C for 30 min, which retained specific neutralizing activity to SARS-CoV-2, potentially useful for passive immunization against coronavirus infection threats as an additive approach to the vaccination. This strategy, as a supportive approach to the vaccination, could also be applicable for directly reducing the effect of COVID-19 infection in gastrointestinal tract, supporting mucosal immunity.

## Introduction

In the global COVID-19 pandemic, immunological studies have proved that antibodies are the effective molecules for sanitizing the body from viruses. However, the formation of novel SARS-CoV-2 mutations is causing a decreased effectiveness of approved vaccines. Moreover, the slow rate of massive vaccination process, due to poor public acceptance and/or insufficient vaccine supplies in some countries, is one of the main factors for continuous reemergence of new virus variants of concern (VOC). The development and registration of new vaccines against constantly emerging mutations require additional time and funding. This underlies to explore new opportunities to establish a stable herd immunity, focusing on the development of highly effective neutralizing antibodies (nAbs) (1). Apparently, nAbs against VOC can be quickly obtained by the vaccination of farm animals with the emergency use-approved (EUA) human vaccines, covering the new mutations of importance. Thanks to mucosal immunity, immune milk with active nAbs could also be theoretically effective and applicable for diminishing SARS-CoV-2 during the course of infection, due to the evidence that oral preparations based on bovine IgG remained stable in the gastrointestinal tract (2). Furthermore, angiotensin-converting enzyme 2 (ACE2) and supportive protease, important for SARS-CoV-2 infection, are both expressed by cells of the entire gastrointestinal tissues. This is also supported by the clinical evidences of orally administered bovine IgG activity in prevention of both upper respiratory and gastrointestinal tract infections (3). It is known that the SARS-CoV-2 infection also causes the gastrointestinal disturbance along with most commonly observed pulmonary symptoms.

The idea to study and validate if vaccinated farm animal milk contains nAbs or not came from the evidence that antibodies to SARS-CoV-2 were found in the milk of lactating women who had COVID-19 or been vaccinated (4). We also reviewed previous publications on the possible benefit from the passive immunization using milk of vaccinated cow. Jawhara (5) first suggested that microfiltered raw immune milk or colostrum collected from SARS-CoV-2-vaccinated cows could provide short-term protection against SARS-CoV-2 infection in humans. Further, Arenas et al. (6) proposed the use of heterologous passive immunity, using Bovine Coronavirus (BCoV) immune milk as an immunostimulant therapy to control SARS-CoV-2 infection, because vaccination of farm animals is well-known and has been described in the literature to protect animals from viruses, including BCoV (7). Gallo et al. (8) reviewed the antiviral properties of native and chemically modified whey proteins and their potential applications in human health, focusing on their application in prevention and treatment of SARS-CoV-2 infection.

However, either the detection of nAbs in the serum and milk of vaccinated household farm animals using EUA human vaccines, or the effect of pasteurization of such immune milk on the SARS-CoV-2 virus-neutralizing activity, has not yet been experimentally validated. To address these important open questions, we performed a series of pilot experiments in 60 milk and serum samples of cows and goats vaccinated with the ZF-UZ-VAC2001 recombinant SARS-CoV-2 human vaccine, containing a dimeric form of the receptor-binding domain (RBD) as the antigen (9). We used this particular vaccine because it had successfully passed a third phase clinical trial in Uzbekistan and was approved for the massive vaccination of the population (10). This vaccine has also been approved for emergency use in China, Indonesia, and Columbia (11).

The ZF-UZ-VAC2001 vaccine has shown its effectiveness against the diverged variants of the SARS CoV-2 virus (10, 12), namely Alfa (92.93%), Gamma (100%), Delta (77.47%), and Kappa (90.0%). Although the neutralization activity of currently used human vaccines has shown a decreased effect against the emerging new VOC B.1.1.529 (Omicron) variant (12), the prolonged time between the second and third dose injections of ZF-UZ-VAC2001 (0,1, 2 vs. 0, 1, 5 months) has revealed a better neutralization effect against the Omicron variant (13). Here, we briefly present the first experimental validation report on obtaining immune milk with nAbs against SARS-CoV-2 from cows and goats after vaccination with RBD-based recombinant protein subunit human vaccine, ZF-UZ-VAC2001 (or ZF2001 or Zifivax).

## Materials and Methods

### Animal Vaccination

The vaccinated farm animals were kept separately from a herd of cows in “Panaev’s Animal Farms” conditions Karakalpakistan, Republic of Uzbekistan. Thirty-two healthy pregnant cows as well as one dairy cow with feeding calf and one lactating goat were vaccinated with the ZF-UZ-VAC2001-recombinant SARS-CoV-2 human vaccine. Revaccination was carried out intramuscularly after 28 and 56 days, according to the manufacturer’s instructions by analogy with those recommended for use in humans, i.e., 1 dose (25 μg/0.5 ml RBD-specific antigen protein formulated with aluminum hydroxide) per 70 kg of animal weight. The sampling of milk and blood was carried out in the morning. The studies were reviewed and approved by the Ethics Committee of the Republic of Uzbekistan for SARS-CoV-2 research (authorization number 6/6 1449 from 13 October, 2020).

### Preparation of Milk and Colostrum Samples

We used the protocol of Fox et al. (14) for human milk to remove lipids from milk samples. Briefly, the milk samples were centrifuged at 800 g for 15 min at room temperature, fat was removed, and the supernatant was transferred to a new tube. Centrifugation was repeated 2x to ensure the removal of all cells and fat. Skimmed acellular milk was aliquoted and frozen at −80°C until testing. In the case of freezing, the samples were re-cleaned by centrifugation as above, after thawing. To study the influence of pasteurization, three different milk samples were preliminarily heated using the various regimes (62.5–63°C for 30 min; 72°C for 5 min; and 85°C for 5 s) according to the State regulations for pasteurization (N 0281-09). The same protocol was applied to the colostrum samples.

### Virus Neutralization Assays

#### MAGLUMI^®^ SARS-CoV-2 Neutralizing Antibody Assay

MAGLUMI^®^ SARS-CoV-2 Neutralizing Antibody assay chemiluminescence imminoassay (CLIA) was performed using fully-automatic chemiluminescence immunoassay analyzer MAGLUMI series, according to the manufacturer’s instructions for human sera (Snibe Diagnostic, Pingshan, China), at the Scientific and Diagnostical Centre of Laboratory Technology “Defactum Laboratories,” Tashkent, Uzbekistan. Briefly, the milk sample was thoroughly mixed with magnetic microbeads coated with ACE2 antigen and recombinant SARS-CoV-2 S-RBD antigen labeled with ABEI. The mixed content was incubated in a buffered condition. SARS-CoV-2 neutralizing antibody, present in milk samples, competes with ACE2 antigen immobilized on magnetic microbeads for binding recombinant SARS-CoV-2 S-RBD antigen labeled with Amino-Butyl-Ethyl-Isoluminol (ABEI). After precipitation in a magnetic field, the supernatant was decanted, and the pelleted magnetic beads were washed. Subsequently, the Starter 1+2 reagent of the MAGLUMI assay kit (Snibe Diagnostic, Pingshan, China) was added to initiate a chemiluminescent reaction. The light signal was measured by a photomultiplier as relative light units (RLUs), which is inversely proportional to the concentration of SARS-CoV-2 neutralizing antibody present in the milk sample. The analyzer automatically calculated the concentration in each sample by means of a calibration curve, which was generated by a 2-point calibration master curve procedure. The neutralization activity of 0.300 μg/ml has provided 50% inhibition of viral growth.

### SARS-CoV-2 Neutralization Test

The NT test was performed in a laboratory with a BSL3 security level of the Virology Centre of the Medical University of Vienna, Austria according to the protocol Koblischke et al. (15). Briefly, 2-fold serial dilutions of heat-inactivated samples were incubated with 50–100 TCID50 SARS-CoV-2 for 1 h at 37°C before the mixture was added to Vero E6 cell monolayers (starting dilution of samples 1:10). Incubation was continued for 2–3 days. NT titers were expressed as the reciprocal of the sample dilution required for 100% protection against virus-induced cytopathic effects. NT titer ≥ 10 was considered positive.

### Determination of Bovine Anti-RBD and Anti-S-Protein-Specific IgG, IgA, and IgM

The determination of antigen-specific antibody isotypes to the receptor-binding domain (RBD) of spike (S) protein and full-length S protein in bovine serum and milk samples was performed by enzyme-linked immunosorbent assay (ELISA). Aliquots of 2 μg/ml of RBD of spike (S) protein or full-length S protein (Genscript, Leiden, Netherlands) were coated overnight onto NUNC Maxisorp 96 well plates (Thermofisher Scientific, Massachusetts, United States). After washing three times with PBST buffer and blocking with 2% BSA (bovine serum albumin) solution, the plates were incubated at 4°C overnight. Serum or milk dilutions (1:500/1:50/1:10 in 0.5% BSA) in amounts of 100 μl/well were added after removing the blocking solution, and plates were stored overnight at 4°C again.

The plates were washed three times with 200 μl PBST. Further, 50 μl of peroxidase-labeled sheep anti-bovine IgG, IgM, or IgA Ab (Bio Rad, Hercules, California, United States) diluted in dilution buffer 1:25,000 was added per well and incubated at room temperture for 120 min with flowing five times washing steps. The color reaction was started by adding 50 μl/well of substrate solution [200 mg 2,2’-azino-bis (3-ethylbenzothiazoline-6-sulfonic acid) (ABTS; Sigma-Aldrich, Darmstadt, Germany)] in 200 ml citric buffer (61.5 mM citric acid, 77.3 mM Na_2_HPO_4_ × 2 H_2_O, pH 4, and 20 μl hydrogen peroxide). The optical density (OD) values corresponding to the levels of antigen-specific antibodies were determined at 405 and 492 nm in an TECAN Infinite F5 ELISA reader. All determinations were performed in duplicates, and the results are shown as mean values with a variation of <5%.

### Statistical Analyses

Data analysis and statistical significance of correlations were performed using Pearson Correlation – Free Statistics Software (16). We computed Pearson correlation including Pearson product moment correlation, covariance, determination, and the correlation *t*-test determining statistical significance from the two-sided *p*-values. The Jarque-Bera and Anderson-Darling normality tests were applied to variables. Significant differences between groups were calculated using one-way analysis of variance (ANOVA) followed by Bonferroni *post hoc* test, and *p* ≤ 0.05 was considered statistically significant (Supplementary Tables 1, 2). The box plots were generated using GraphPad Prism 8 (GraphPad Software Inc.).

## Results and Discussion

We performed vaccination of farm animals (lactating cow and goat) with revaccination carried out after 28 and 56 days. Examination of the udder, palpation of the lymph nodes, mucous membranes, and measurement of rectal temperature, as well as the observation of the behavior of animals did not reveal any side effects during daily monitoring of animals after vaccination.

Since the first dose vaccine injection period, we regularly collected and evaluated the virus neutralization activity of the immune milk and blood serum of the lactating cow and goat, measuring the inhibition level of viral RBD binding to the ACE2 receptor (15).

We detected the neutralizing antibodies to the RBD domain and S-protein of SARS-CoV-2 in the blood serum of a vaccinated cow (*n* = 1) as early as 2 weeks after the first vaccination. Results showed that revaccination contributed to an increase in the effect of inhibition. We noted maximum neutralization activity of blood serum (100%) and milk (40%) on the 77th day from the date of the first vaccination. The correlation between neutralization rate of cow sera and milk during the entire course of vaccination was significant at *r* = 0.96, *p* = 0.044.

Complete viral neutralization of the cytopathic effect on Vero E6 cells was detected even with 120-fold serum titration. The blood serum and milk of the vaccinated cow contained specific IgG to the RBD domain and S-protein of SARS-CoV-2. We also found statistically significant correlation between the level of IgG specific to the RBD domain and the neutralization rate of milk of vaccinated cows (*r* = 1.0, *p* = 0.002, Supplementary Table 1).

**FIGURE 1:**
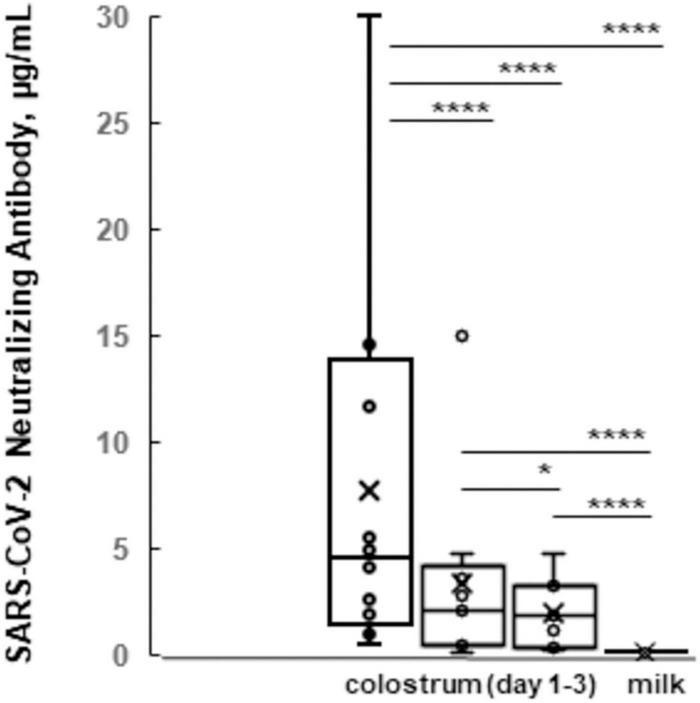
The box plots of SARS-CoV-2 neutralizing antibody levels of colostrum vs. milk. Average indices (y-axis) for immune colostrum during first 3 days and immune milk samples (x-axis) are shown. *p ≤ 0.05, and ****p ≤ 0.0001; ns, not statistically significant difference.

Further, we vaccinated a group of 22 “Simmental,” nine “Holstein Friesians,” and one “Brown Swiss” cows during the dry period of the third trimester of pregnancy (Supplementary Table 3). After calving (*n* = 32), we determined that that the colostrum of the first day had a greater ability to neutralize the SARS-CoV-2 virus (4.080 μg/ml) compared to colostrum of the subsequent days (2.106, 1.960, and 1.126 μg/ml) and milk (0.222 μg/ml; Figure 1). These indices were above or within the range of 50% neutralization activity (0.30 μg/ml), demonstrating an efficiency of immune colostrum and milk of our experiment.

In another pilot experiment in a domestic lactating goat (*Capra hircus*), it was sufficient to use only two-dose injection of the vaccine to achieve the effect of immunization and obtain immune milk samples. The correlation between neutralization rate of goat sera and goat milk during the entire course of vaccination was high at *r* = 0.99 and *p* = 0.006 (Supplementary Table 1). The maximum of virus-neutralizing activity of the immune goat milk was 1.486 μg/ml on the 14th day of the third dose vaccine injection.

We further investigated the possibility of transferring of SARS-CoV-2-specific IgG antibodies via milk from cow to its milking calf. Toward this goal, we performed the analysis of sera from calf during active vaccination of cow. Results showed that there was statistically significant correlation (*r* = 0.95, *p* = 0.05, Supplementary Table 1) between the level of IgG specific to RBD of vaccinated cow and calf fed by milk of vaccinated mother cow. This showed the possibility of passive immunization using milk of vaccinated mother cow, containing specific IgG against SARS-CoV-2.

In perspectives, as highlighted above (2–8), there is a huge opportunity to use the passive immunization property of immunized cow milk in humans. For this, there is a need for determining the optimal pasteurization conditions of immune milk to keep its biologically active neutralization against SARS-CoV-2. Toward this goal, we have studied several pasteurization regimes of immunized milk while analyzing its neutralization activity of SARS-CoV-2. We tested 20 milk samples obtained from vaccinated cow and goat, pasteurizing them at different temperatures and regimes of pasteurization according to State regulations (62.5–63°C for 30 min, 72°C for 5 min, and 85°C for 5 s).

**FIGURE 2:**
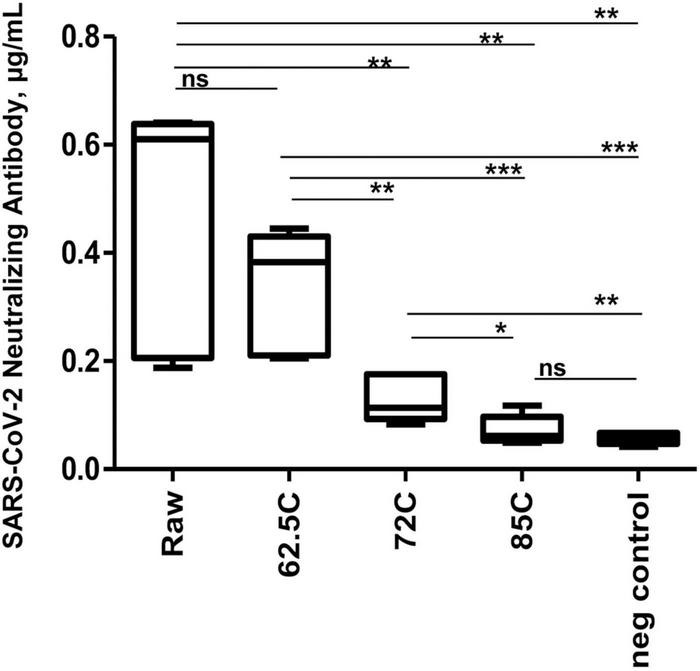
The box plots of SARS-CoV-2 neutralizing antibody levels upon milk pasteurization. Average indices (y-axis) for before pasteurization (raw) and pasteurized immune milk samples at the different regimes of pasteurization (x-axis) are shown. *p ≤ 0.05, **p ≤ 0.01, and ***p ≤ 0.001; ns, not statistically significant difference. Negative control represents milk sample from non-vaccinated animal.

As expected, temperature treatment for pasteurization of milk product negatively correlated with neutralization activity (*r* = −1.0, *p* = 0.05). Compared to before-pasteurization raw milk (0.5 ± 0.2 μg/ml), pasteurization treatments at 72°C (for 5 min) and 85°C (for 5 s) temperatures showed sharp activity decreases (0.1 ± 0.04 and 0.05 ± 0.01 μg/ml, respectively). However, the pasteurization at 62.5–63°C during 30 min retained sufficiently active neutralization property in nAbs chemiluminescence immunoassay (0.3 ± 0.1 μg/ml), while a small decrease observed between raw and pasteurized immune milk at 62.5–63°C during 30 min was statistically non-significant (*p* = 0.24; Figure 2 and Supplementary Table 2).

Our first experimental validation results indicated that the ZF-UZ-VAC2001 human vaccine can be safely used for the vaccination of household farm animals to obtain immuno-biologically active milk and serum, containing specific nAbs against SARS-CoV-2. There is no need for the substantial corrections in dose calculations for vaccination of the animals, which can be performed in accordance to the manufacturer’s instructions and protocols for human vaccination. Interestingly, we experimentally observed that milk and serum of vaccinated cow not only have active neutralization property against SARS-CoV-2 but also biologically active specific IgG antibodies to RBD and S protein, transferred from mother cow’s milk to its milking calf, passively forming offspring’s immune protection. We found that the most optimal condition for immunized milk pasteurization can be achieved 62.5–63 °C during 30 minutes. This pasteurization condition retains efficient virus neutralizing antibody activity against SARS-CoV-2, providing future opportunity for the passive immunization of human by milk consumption.

## Limitations

The limitation of this study is a small number of farm animals (one lactating cow and goat, and 32 pregnant cows) included for vaccination experiments. There is a need for future large-scale experiments, using an increased number of farm animals and testing the presence of nAbs over a period after vaccination and feeding with colostrum, which is in progress in our center.

## Conclusion

The results collectively showed that pasteurized immune milk obtained from vaccinated household animals using human vaccine against SARS-CoV-2, administered herein, potentially could be useful for passive immunization against coronavirus infection threats. The use of immune colostrum, milk, and dairy products with neutralizing antibodies from vaccinated cows and goats seems to be a promising approach for the preparation of safe and natural prophylactic agents against human and animal infections. Moreover, immune milk could be useful to diminish the effect of SARS-CoV-2 supporting mucosal immunity. Therefore, it may be as an additive treatment option during COVID-19 infection. However, the immune milk strategy, proposed herein, does not substitute the necessity of vaccination.

## Data Availability Statement

The raw data supporting the conclusions of this article will be made available by the authors, without undue reservation.

## Ethics Statement

The animal study was reviewed and approved by Ethics Committee Republic of Uzbekistan (authorization number 6/6 1449 from 13.10.2020). Written informed consent for participation was not obtained from the owners because Verbal consent was favored by the owner of “Panaev’s Animal Farm” over written agreement.

## Author Contributions

VG and IA: project administration, conceptualization, methodology, investigation, data curation, and writing—original draft, review and editing. FG: consultation, methodology, and writing—review and editing. YL and ST: methodology, investigation, and writing—review and editing. SK, GB, KK, MR, NM, and AB: investigation, data collection, and curation. All authors contributed to the article and approved the submitted version.

## Conflict of Interest

The authors declare that the study concept and results are filed for patenting at the Intellectual Property Agency under the Ministry of Justice of the Republic of Uzbekistan with pending applications No. IAP 2021 0365 and IAP 2022 0054. The reviewer DM declared a shared affiliation with the author VG to the handling editor at the time of review.

## Publisher’s Note

All claims expressed in this article are solely those of the authors and do not necessarily represent those of their affiliated organizations, or those of the publisher, the editors and the reviewers. Any product that may be evaluated in this article, or claim that may be made by its manufacturer, is not guaranteed or endorsed by the publisher.
