# Supplementary material for: Milk of Cow and Goat, Immunized by Recombinant Protein Vaccine ZF-UZ-VAC2001(Zifivax), Contains Neutralizing Antibodies Against SARS-CoV-2 and Remains Active After Standard Milk Pasteurization
Source: Front Nutr. 2022 Jun 13;9:901871. doi: 10.3389/fnut.2022.901871 (PMC9249723; doi:10.3389/fnut.2022.901871)
Supplement: Supplementary file 1 [file Table_1.docx]

**Supplementary Table 1.** Statistical analysis of correlations between parameters (n=60).

| **Parameters** | **Correlation** | **p-value (2 sided)** |
| --- | --- | --- |
| Neutralization rate of cow sera vs milk in during vaccination | 0.96 | 0.044 |
| IgG-RBD vs neutralization rate of milk | 1.00 | 0.002 |
| IgG-RBD of sera after active vs passive immunization | 0.95 | 0.053 |
| Temperature of milk pasteurization vs neutralization rate | -1.00 | 0.045 |
| Neutralization rate of goat sera vs milk in during vaccination | 0.99 | 0.006 |
| Neutralization rate of cow milk vs goat milk in during vaccination | 0.98 | 0.023 |
