# Supplementary material for: Milk of Cow and Goat, Immunized by Recombinant Protein Vaccine ZF-UZ-VAC2001(Zifivax), Contains Neutralizing Antibodies Against SARS-CoV-2 and Remains Active After Standard Milk Pasteurization
Source: Front Nutr. 2022 Jun 13;9:901871. doi: 10.3389/fnut.2022.901871 (PMC9249723; doi:10.3389/fnut.2022.901871)
Supplement: Supplementary file 2 [file Table_2.docx]

**Supplementary Table 2.** Comparisons of nAbs levels of immune milk at different pasteurization conditions (n=20); shown are the corresponding p-values.

| sample | raw | 62.5ºC | 72ºC | 85ºC | neg control |
| --- | --- | --- | --- | --- | --- |
| raw | – | 0.2405 | 0.0069 | 0.0026 | 0.0020 |
| 62.5ºC | 0.2405 | – | 0.0030 | 0.0004 | 0.0003 |
| 72ºC | 0.0069 | 0.0030 | – | 0.0139 | 0.0035 |
| 85ºC | 0.0026 | 0.0004 | 0.0139 | – | 0.2903 |
| neg control | 0.0020 | 0.0003 | 0.0035 | 0.2903 | – |
