# Supplementary material for: Milk of Cow and Goat, Immunized by Recombinant Protein Vaccine ZF-UZ-VAC2001(Zifivax), Contains Neutralizing Antibodies Against SARS-CoV-2 and Remains Active After Standard Milk Pasteurization
Source: Front Nutr. 2022 Jun 13;9:901871. doi: 10.3389/fnut.2022.901871 (PMC9249723; doi:10.3389/fnut.2022.901871)
Supplement: Supplementary file 3 [file Table_3.docx]

**Supplementary Table 3.** Information on cows used for vaccination and injected doses.

| **#** | **ID number** | **Cow breed** | **Age,** | **Weight,** | **Pregnancy** | **Vaccination** | **Booster** | **Booster** | **Total** |
| --- | --- | --- | --- | --- | --- | --- | --- | --- | --- |
|  |  |  | **years** | **kg** | **number at vaccination** | **doses*** | **doses** | **doses** | **doses** |
| 1 | 0000 | Simmental | 6 | 689 | 4 | 10 | 10 | 10 | 30 |
| 2 | 50728 | Simmental | 6 | 720 | 3 | 10 | 10 | 10 | 30 |
| 3 | 29650 | Simmental | 6 | 735 | 4 | 10 | 10 | 10 | 30 |
| 4 | 6094 | Simmental | 5 | 730 | 3 | 10 | 10 | 10 | 30 |
| 5 | 2651 | Simmental | 6 | 724 | 4 | 10 | 10 | 10 | 30 |
| 6 | 3862 | Simmental | 5 | 732 | 3 | 10 | 10 | 10 | 30 |
| 7 | 9642 | Simmental | 6 | 715 | 3 | 10 | 10 | 10 | 30 |
| 8 | 1795 | Holstein Friesians | 6 | 592 | 4 | 8 | 8 | 8 | 24 |
| 9 | 30639 | Simmental | 6 | 725 | 4 | 10 | 10 | 10 | 30 |
| 10 | 67431 | Simmental | 6 | 720 | 4 | 10 | 10 | 10 | 30 |
| 11 | 1778 | Holstein Friesians | 6 | 569 | 4 | 8 | 8 | 8 | 24 |
| 12 | 7 | Simmental | 4 | 581 | 2 | 8 | 8 | 8 | 24 |
| 13 | 12604 | Simmental | 6 | 574 | 4 | 8 | 8 | 8 | 24 |
| 14 | 4011 | Simmental | 5 | 716 | 3 | 10 | 10 | 10 | 30 |
| 15 | 7806 | Holstein Friesians | 5 | 582 | 3 | 8 | 8 | 8 | 24 |
| 16 | 6938 | Holstein Friesians | 5 | 564 | 3 | 8 | 8 | 8 | 24 |
| 17 | 4802 | Holstein Friesians | 6 | 575 | 3 | 8 | 8 | 8 | 24 |
| 18 | 9184 | Simmental | 6 | 724 | 3 | 10 | 10 | 10 | 30 |
| 19 | 8673 | Holstein Friesians | 6 | 585 | 4 | 8 | 8 | 8 | 24 |
| 20 | 0261 | Brown Swiss | 4 | 440 | 2 | 6 | 6 | 6 | 18 |
| 21 | 3859 | Simmental | 5 | 727 | 3 | 10 | 10 | 10 | 30 |
| 22 | 76300 | Simmental | 6 | 710 | 4 | 10 | 10 | 10 | 30 |
| 23 | 00220 | Holstein Friesians | 4 | 600 | 2 | 8 | 8 | 8 | 24 |
| 24 | 67430 | Simmental | 6 | 698 | 4 | 10 | 10 | 10 | 30 |
| 25 | 05899 | Simmental | 6 | 716 | 4 | 10 | 10 | 10 | 30 |
| 26 | 0154 | Simmental | 5 | 720 | 3 | 10 | 10 | 10 | 30 |
| 27 | 0779 | Holstein Friesians | 5 | 735 | 3 | 10 | 10 | 10 | 30 |
| 28 | 0012 | Simmental | 4 | 587 | 2 | 8 | 8 | 8 | 24 |
| 29 | 3957 | Simmental | 6 | 725 | 4 | 10 | 10 | 10 | 30 |
| 30 | 6680 | Simmental | 6 | 698 | 4 | 10 | 10 | 10 | 30 |
| 31 | 0004 | Simmental | 4 | 600 | 2 | 8 | 8 | 8 | 24 |
| 32 | Astery | Holstein Friesians | 3 | 500 | 2 | 7 | 7 | 7 | 21 |
| 33 | 75822 | Simmental | 5 | 650 | 3 | nv | nv | nv | nv |
| 34 | 7842 | Simmental | 6 | 625 | 4 | nv | nv | nv | nv |

*Note: nv: non-vaccinated, negative control cows; one dose contained 25µg/0.5ml RBD-specific antigen protein formulated with aluminum hydroxide.
